# Photocrosslinked Dextran-Based Hydrogels as Carrier System for the Cells and Cytokines Induce Bone Regeneration in Critical Size Defects in Mice

**DOI:** 10.3390/gels4030063

**Published:** 2018-07-20

**Authors:** Ulrike Ritz, Marc Eberhardt, Anja Klein, Petra Frank, Hermann Götz, Alexander Hofmann, Pol Maria Rommens, Ulrich Jonas

**Affiliations:** 1 Biomatics Group, Department of Orthopaedics and Traumatology, University Medical Center of the Johannes Gutenberg University Mainz, 55131 Mainz, Germany; marc.eberhardt@unimedizin-mainz.de (M.E.); anja.klein@unimedizin-mainz.de (A.K.); hofmann.trauma-surgery@gmx.net (A.H.); pol.rommens@unimedizin-mainz.de (P.M.R.); 2 Macromolecular Chemistry, Department Chemistry Biology, University of Siegen, 57076 Siegen, Germany; frank@chemie.uni-siegen.de (P.F.); jonas@chemie.uni-siegen.de (U.J.); 3 Biomatics Group, Platform Biomaterials, University Medical Center of the Johannes Gutenberg University Mainz, 55131 Mainz, Germany; hgoetz@uni-mainz.de

**Keywords:** dextran-based hydrogels, carrier system, SDF-1, bone regeneration, critical size defect

## Abstract

Modified biomaterials have for years been the focus of research into establishing new bone substitutes. In our preceding in vitro study employing different cell cultures, we developed chemically and mechanically characterized hydrogels based on photocrosslinkable dextran derivatives and demonstrated their cytocompatibility and their beneficial effects on the proliferation of osteoblasts and endothelial cells. In the present in vivo study, we investigate photocrosslinked dextran-based hydrogels in critical size defects in mice to evaluate their potential as carrier systems for cells or for a specific angiogenesis enhancing cytokine to induce bone formation. We could demonstrate that, with optimized laboratory practice, the endotoxin content of hydrogels could be reduced below the Food and Drug Administration (FDA)-limit. Dextran-based hydrogels were either loaded with a monoculture of endothelial cells or a co-culture of human osteoblasts with endothelial cells, or with stromal-derived-growth factor (SDF-1). Scaffolds were implanted into a calvarial defect of critical size in mice and their impact on bone formation was assessed by µCt-analyses, histology and immunohistology. Our study demonstrates that promotion of angiogenesis either by SDF-1 or a monoculture of endothelial cells induces bone regeneration at a physiological level. These in vivo results indicate the potential of dextran-based hydrogel composites in bone regeneration to deliver cells and cytokines to the defect site.

## 1. Introduction

Although increasing knowledge about fracture healing has been gained in the last decades for bone defects of critical sizes, many mechanistic details of healing are still unknown. These medical conditions represent a general challenge for surgeons and no agreement exists on an optimal therapeutic strategy. Autologous bone grafting is commonly performed, yet this method often inflicts donor graft morbidity with consequences such as pain, stress and surgical revision [1]. Moreover, the volume of autologous bone graft that can be salvaged is tightly limited. Alternative approaches are either based on pharmacological methods, for example the administration of cytokines or growth factors in the bone defect, or they involve living cells (like cell-based tissue engineering) [2]. Many studies demonstrated that besides induction of bone growth a critical point in bone tissue engineering is the early supply of implant-associated cells and tissue with nutrients and oxygen, which requires vascularization of the implant material and its surrounding region. During the last decade, substantial research has been conducted in this field but continuous optimization of existing therapeutic approaches is required [3,4]. In view of an advanced therapy, many facets need to be considered, specifically the following three points:

1. The material aspect—the choice of classical implant materials like steel or titanium versus new materials like polymers or composites.
2. The best molecular cue to induce vascularization, as well as bone growth.
3. Loading or immobilization of cytokines or growth factors in the material versus prevascularization of the implant by cell-based tissue engineering.

Biomaterials are defined as nonviable materials used in a medical device, intended to interact with biological systems [5]. Hydrogels, based on different polymers, are water-swollen networks that represent a subclass of biomaterials, whose role in biomaterial research rose extremely during the last decade [6,7,8]. They represent an attractive option as carrier systems in medical applications as they resemble key aspects of the extracellular matrix [9,10,11]. Depending on their specific chemical nature, hydrogels can demonstrate all characteristics essential for a biomaterial, such as being degradable, biocompatible and not cytotoxic, while their stability can be tailored by chemical design [12]. Polymers of biological origin, like alginate, κ-carrageenan and hyaluronic acid have been successfully used as starting material for cross-linked hydrogels in a biomedical context [13,14,15,16,17]. Bioactive hydrogels synthesized from different materials have been tested with various cells for their application in bone tissue engineering [18,19,20]. We could demonstrate in vitro that photocrosslinked polysaccharide hydrogels synthesized from pullulan, amylose and dextran support cell growth and differentiation of primary human osteoblasts as well as of endothelial cells [21]. In that in vitro study the dextran-based hydrogels demonstrated the best results concerning cellular experiments. Moreover proteins (cytokines, growth factors inducing the desired function) can be integrated into the hydrogel matrix while retaining their biological functionality [22]. Examples of suitable growth factors for immobilization in hydrogels are bone morphogenic proteins [23], which induce bone formation, or stromal-derived-growth factor (SDF-1) or vascular endothelial growth factor (VEGF) to induce neo-vascularization and angiogenesis [24]. 

One potential problem concerning implant materials and their biocompatibility is their contamination with endotoxins. Endotoxins are lipopolysaccharides (LPS), which are located on the outer cell membrane of Gram-negative bacteria. They are ubiquitously present in the environment and induce a toxic response when entering the blood stream. They are recognized by innate immune cells, thus activating the respective infectious pathways. An endotoxin contamination in humans results in infection and inflammation, which ultimately can lead to multiple organ failure and eventually to death. Subsequent removal of endotoxin from contaminated biomaterials is very difficult, thus avoiding endotoxin contamination during the whole process chain from synthesis to implantation is highly advised [25,26,27]. For this reason, we determined endotoxin contamination and optimized the laboratory processes for the hydrogel materials used in this study.

In our previous cell culture studies, we developed and tested various polysaccharide hydrogels for their mechanical and swelling properties, cytotoxicity, cytokine immobilization, release and function, cell growth, proliferation and marker gene expression after different durations. We demonstrated in vitro that polysaccharide hydrogels, especially based on dextran, showed a great potential for use as biomaterial in tissue engineering [21,24]. Studies by other groups confirmed that dextran-based hydrogels can be used in medical applications, for example as wound dressings [28,29]. However, to our knowledge no in vivo study exists showing the potential of dextran-based hydrogels as bone substitutes or carrier system to induce bone regeneration in critical size defects. In contrast to many of the polysaccharides used in a biomedical context, like chitosan, alginate and hyaluronic acid, which contain besides hydroxyl moieties various functional groups (for example amine or carboxylic acid substituents) with characteristic reactivity, dextran comprises only hydroxyl groups. Dextran represents a highly biocompatible base material, in which the desired functionality can be introduced by specific chemical modifications to result in tailored, dextran-based hydrogels [30].

Beyond the positive results previously obtained by in vitro cell culture studies with dextran-based hydrogels, it now has to be demonstrated that these materials can unfold their positive characteristics in an in vivo model for bone regeneration. For this purpose, we employed an established model of critical size defects in the calvaria of mice to investigate the potential of these photocrosslinked dextran-based hydrogels to induce bone formation and fracture healing by µCt analyses, as well as histological staining. As a further important aspect, the present study provides a comparison between the cell-based approach of bone tissue engineering on the one hand and a growth factor-based strategy on the other. The cell-based approach is more time- and cost intensive compared to the application of growth factors and thus would be competitive only if it was significantly more effective.

## 2. Results and Discussion

### 2.1. Synthesis of Dextran-Based Hydrogels and Immobilization of Growth Factor

Dextran-based hydrogels as 3D scaffolds were prepared as described in the materials and methods section. Gelatin and silica particles were included in the hydrogel matrix to increase mechanical stability and to enhance biocompatibility as described before [21,24]. Characterization of swelling behaviour, degradation properties and mechanical stability were analysed in our former studies [21,24] as well as by others [31]. The composite hydrogels demonstrated the expected stability and swelling behaviour. Moreover, we could demonstrate in these studies that human primary osteoblasts as well as endothelial cells both grow and proliferate on these dextran-based hydrogels [21]. Hence, they were selected for the present in vivo experiments. The cytokine SDF-1 was chosen for its known proangiogenic effects [32,33]. In addition, we and others could demonstrate that SDF-1 keeps its functional activity after immobilization in dextran hydrogels [21,34]. Our former in vitro study demonstrated that immobilization of SDF-1 and bone morphogenic protein 2 (BMP-2) via EDC/NHS-coupling resulted in a delayed release compared to immobilization without EDC/NHS. Without EDC/HNS coupling 90% of the growth factors were liberated within eight hours, whereas with EDC/NHS coupling the release was prolonged to thirty hours [21]. This release kinetic is consistent with literature reports and should be adequate to induce bone regeneration [35]. In the present in vivo study, BMP-7 was selected as a positive control, as it has been shown to promote bone formation in vivo to a non-physiological extent [36,37]. BMP-7 was used as model cytokine to induce augmented bone formation, which may not be of relevance for a practical clinical application but which allows unambiguous evaluation of its efficiencies under the present experimental conditions (maximum effect positive control). 

It is known that dextran is enzymatically degraded by dextranase, an enzyme existing in mammalian tissues [38]. Moreover, it has been demonstrated that incorporation of functional groups affects the characteristics of hydrogels. For example, immobilization of angiogenic factors—like VEGF—lead to a more rapid degradation of dextran hydrogels and to a faster release of the bound factors [28,38].

Besides defining the effect of prevascularized scaffolds on bone formation, we also wanted to compare the extent of enhanced bone formation between the two strategies of (1) cell seeding (mono-culture endothelial cells and co-culture of endothelial and osteoblastic cells) versus (2) matrix-modification with a growth factor demonstrating positive effects on angiogenesis (SDF-1). In the former in vitro study, we demonstrated an even distribution of the cells (human osteoblasts as well as human umbilical vein endothelial cells (HUVEC)) in the dextran-based hydrogels [21].

### 2.2. Endotoxin Assay

Endotoxin contamination is currently discussed as an underestimated problem, especially in the preparation and application of biomaterials [39]. With this issue in mind we tested endotoxin contamination of our photocrosslinked dextran-based hydrogels prior to implantation in mice to avoid any endotoxin-related complications and host response. Our aim was to only implant materials with an endotoxin contamination below the FDA (Food and Drug Administration) limit of 0.5 EU/mL. Endotoxin contamination was evaluated with a standard limulus amebocyte lysate (LAL) assay [40]. Beside the LAL assay to test endotoxin contamination, other cellular based assays exist [27]. However, as the LAL assay is the most established one, we chose this test for our studies. 

Initial testing by LAL assay demonstrated a mean value of 2.62 EU/mL (standard deviation (SD) 0.053) endotoxin concentration in the hydrogel disks prepared in 96 well plates two weeks after production in a standard chemical laboratory without optimization of laboratory procedures, which is approximately 5 times higher than the maximal recommended FDA limit. One known source of endotoxin contamination is the water supply, which in our case consisted of a Millipore Direct-Q UV 3 filtration device. Comparison of water quality and endotoxin levels between a several-months-old filter set (still producing water with very low ion concentration and standard resistivity of 18.2 MΩ cm) and a freshly installed filter set demonstrated a substantial reduction of endotoxin contamination in the water from above to below FDA limit by filter exchange. Besides ensuring highest water quality, utilizing only newly purchased chemicals as provided by the suppliers for synthetic procedures and wiping of the bench surface with 70% aqueous ethanol solutions directly before hydrogel composite preparation further resulted in a substantial reduction of the endotoxin levels below FDA limit for the final hydrogel disks (Table 1).

We were able to reduce endotoxin contamination and concentration by optimizing laboratory procedures. Based on this evidence, endotoxins are underestimated in their biological effects especially concerning biomaterials [39]. This aspect needs to be kept in focus and all (bio) materials should be analysed for endotoxin contamination before starting in vivo experiments.

### 2.3. Calvarial Critical Size Defect (CSD) Model in Mouse

In order to assess in vivo the synthesized hydrogels, either incubated with cells or modified with a growth factor, a calvarial defect model of critical size (CSD) in mice was chosen. The calvarial defect model is one of the most commonly employed standard models to evaluate bone regeneration [41]. Advantages of this procedure are the possibility for standardization, high reproducibility and simple evaluation by employing radiological and histological analyses. A possible disadvantage of this approach may be the negligence of biomechanical effects for bone regeneration. Yet, for initial rapid screening of our dextran-based hydrogel material for their performance to induce bone formation and as guiding materials in bone growth we opted for this CSD model. As we transplanted human derived cells into mice, we used athymic nude mice, which is an accepted model for bone regeneration [42,43]. These mice are immunodeficient and lack T-cells. The role of T-cells in bone regeneration is not yet fully understood but some studies describe preservative or even stimulating effects of T-cells on bone regeneration [44,45,46]. Therefore, a lack of T-cells could influence bone regeneration. However, as we compare immunodeficient mice among each other but not with immunocompetent mice, the influence of the employed biomaterial can be unambiguously analysed using this model.

A CSD is defined as the smallest defect in bone that does not heal spontaneously during the lifetime of the animal. It depends on the chosen animal, its age and gender as well as the employed strain [41,47,48]. The size of a CSD in mice is controversially discussed, ranging from 1 mm to 5 mm. After testing a defect size from 1.8 to 3.5 mm in our athymic mice model, we chose a size of 2.7 mm for our experiments, as with this defect size even after 6 months no bone regeneration could be detected in our calvarial model (data not shown). Into this defect we implanted different types of the photocrosslinked dextran-based hydrogel disks: unmodified EBP-CMD (“*e*poxy *b*enzo*p*henone-modified *c*arboxy*m*ethyl *d*extran”) hydrogel [21,24], EBP-CMD hydrogel loaded with a monoculture of HUVECs, EBP-CMD dextran-based hydrogels loaded with a co-culture of HUVECs and osteoblasts (hOBs), EBP-CMD hydrogels with immobilized SDF-1, a negative control without hydrogel and as positive control EBP-CMD hydrogels with immobilized BMP-7 (see experimental section). The chemical composition of the polymer matrix was identical in all groups. 

### 2.4. Bone Volume/Total Volume

**Figure 1:**
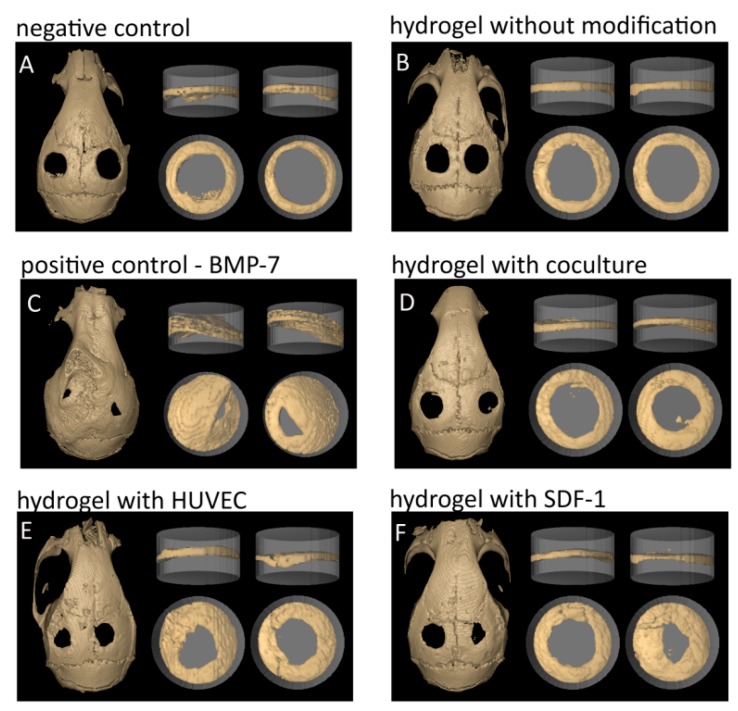
Visual representative presentation of the quantification technique comparing the two control groups to the four hydrogel groups. The grey cylinder (width 3 mm, height 2 mm) corresponds to the defined standard volume of interest representing the total volume (TV). The bone growth inside the cylinder represents the bone volume for the calculation of the ratio bone volume (BV)/ total volume (TV). (**A**) negative control; (**B**) hydrogel without modification; (**C**) BMP-7: positive control; (**D**) hydrogel with coculture; (**E**) hydrogel with HUVEC; and (**F**) hydrogel with SDF-1.

Eight weeks after setting the critical size defect (2.7 mm) the mice were sacrificed and their skulls were fixed and trimmed to fit into tubes for µCt analyses. In order to define and quantify the formation of new bone in the area of hydrogel implantation we determined the ratio of bone volume to total volume by µCt and 3D reconstruction. Figure 1 shows the visual representation of the results from this quantification technique for the four hydrogel groups in comparison to the two control groups. The grey cylinder (width 3 mm, height 2 mm) corresponds to the defined standard volume of interest (total volume/TV), which encloses the total bone defect volume at time of operation. The beige-coloured bone tissue was calculated as a percentage of the entire volume of interest (bone volume/BV).

The 3D reconstructions demonstrate the functionality of our model. The negative control (Figure 1A) shows hardly any bone growth with a defect comparable to the size directly after drilling (2.7 mm). The pure hydrogel (Figure 1B) is comparable to the negative control indicating that the dextran hydrogel alone has no influence on bone regeneration. Only after modification with cells or growth factors an effect can be observed. Dextran-hydrogels seem to act as “guiding layer” inducing more defined bone growth similar to bone of the calvaria. The positive control BMP-7 (Figure 1C) shows an excessive bone formation in all directions, which can be interpreted as unlimited bone growth. In the last years some reports were published describing that BMPs can induce heterotopic ossification, especially when applied in high doses indicating that BMP-7 may not be of clinical relevance. Moreover, the resulting bone presents a structure with extreme pores and low stability [49,50,51]. However, in our model BMP-7 is an excellent positive control. In contrast to this excessive BMP-7-induced effect, a physiological bone regeneration would be preferable, resulting in a bone structure similar to the intact calvaria.

Dextran-based hydrogels loaded with a monoculture of HUVEC cells (Figure 1E) or SDF-1 (Figure 1F) demonstrated the best bone regeneration. Interestingly, a co-culture (Figure 1D) consisting of HUVEC and human osteoblasts demonstrated lower bone regeneration than HUVEC cells alone or hydrogels loaded with SDF-1. This could be due to the fact that in the co-culture only half the number of HUVECs is included compared to the monoculture. It seems that the cell number plays a critical role in tissue regeneration [52]. Changing the hOB/HUVEC ratio from 1:1 to 1:3 and increasing the absolute number of HUVEC could lead to better and faster bone formation. In a former study, we could show that hOB monoculture does not lead to bone formation potentially due to lack of efficient nutrient and oxygen supply [42]. Due to these findings, we did not employ the monoculture osteoblast group in the current study.

**Figure 2:**
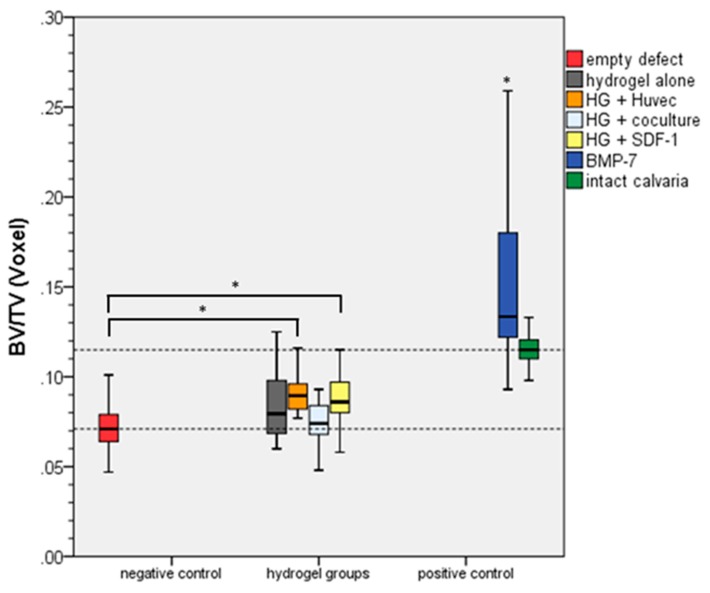
Quantitative evaluation employing the BV/TV method. The positive control groups are significant different to all other groups (*). From the hydrogel groups only the two groups HG-HUVEC and HG-SDF1 demonstrated significant differences compared to the negative control (*, *p* ≤ 0.05).

Figure 2 shows the quantitative evaluation of the results of the BV/TV method presented in Figure 1. It demonstrates that all experimental groups are statistically significant different when compared to the positive groups BMP-7 and intact calvaria. In comparison to the negative control group, only HG-HUVEC and HG-SDF1 demonstrated significant differences.

### 2.5. Histology

**Figure 3:**
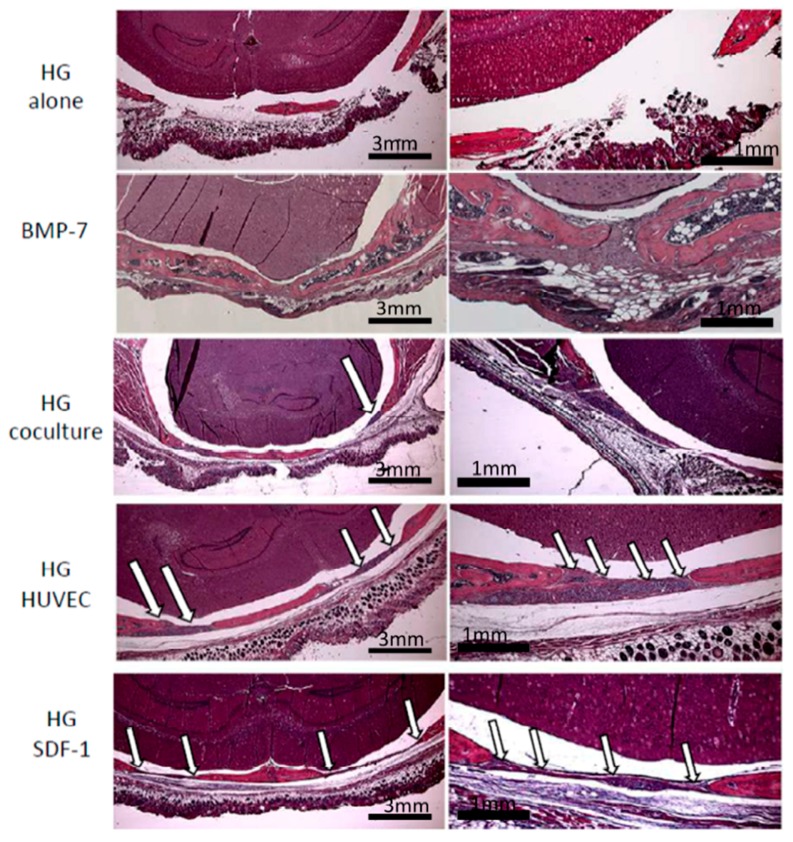
Hematoxylin-eosin (HE) staining of the calvarial defects after treatment with dextran-based hydrogels modified with cells, BMP-7, or SDF-1. Arrows indicate newly formed bony structures and viable cells visible in the HUVEC and SDF-1 groups as well as in the co-culture group, though to a lower extent.

Histologic analysis of the calvarial defects with implanted dextran-based hydrogels modified with cells or SDF-1 confirmed the findings of our volumetric analysis (Figure 3). The positive control (BMP-7) demonstrated an excessive bone growth with trabecular osseous tissue penetrated by red bone marrow. In the group HG alone the full defect is still visible and not even filled with a fibrous membrane structure. The co-culture group is characterized by formation of a thin fibrous membrane containing only few cells and no bony structure. In contrast, mice treated with dextran-based hydrogels loaded with immobilized SDF-1 or a monoculture of endothelial cells demonstrated considerable signs of bone regeneration. The fibrous membrane structure is complemented by a thin bony structure covering the whole defect. Moreover, active cells and areas of ossification were clearly present (Figure 3, indicated by arrows).

**Figure 4:**
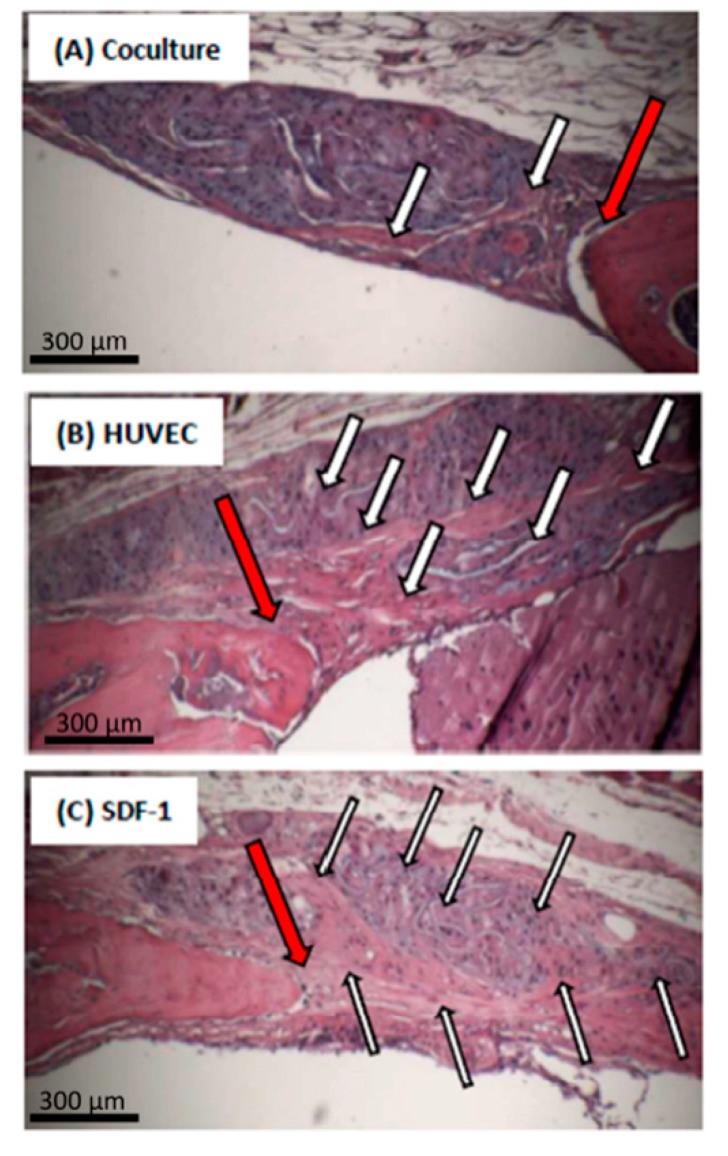
Hematoxylin-eosin (HE) staining of the calvarial defect region: zoom into the transitional region between native bone and new formed bone in the groups co-culture (**A**); monoculture of endothelial cells (**B**) and SDF-1 (**C**). Red Arrows indicate the transitional regions, black/white arrows indicate active cells and areas of new ossification.

Figure 4 focuses on the margin between native bone and new formed bone in the groups co-culture, SDF-1 and monoculture of endothelial cells. Arrows indicate active cells and areas of ossification, which are higher in the SDF-1 and HUVEC-group compared to the co-culture group. Overall, the results of the HE-staining confirm the µCt-analyses and the bone volume/total volume quantification.

No residues of dextran-based hydrogels could be detected by HE-staining. This is in accordance to literature, where it is described that modified dextran-based hydrogels are biodegraded in mammalian tissues [53].

**Figure 5:**
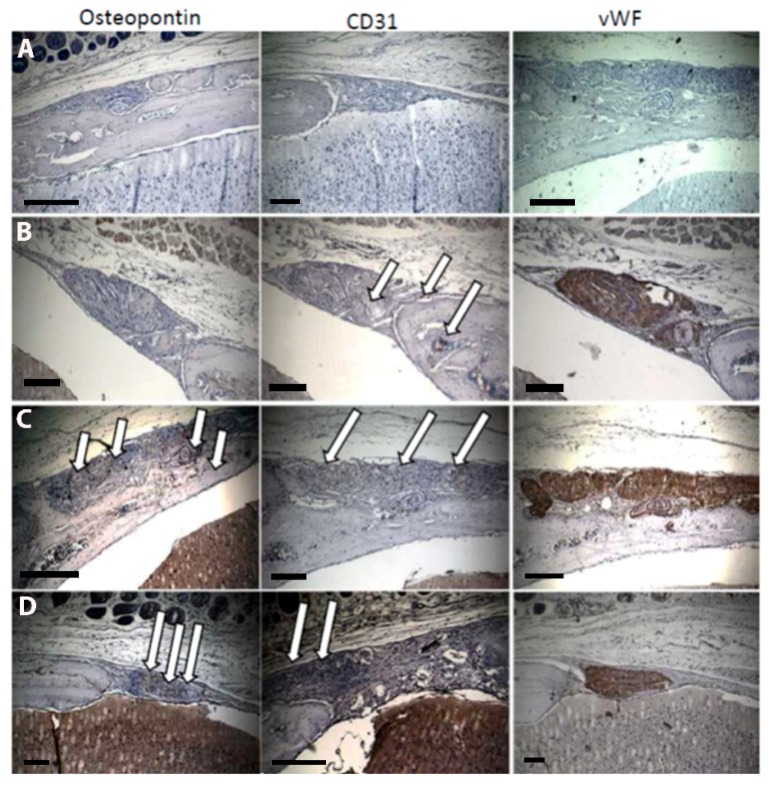
Immuohistological stainings for the endothelial markers von Willebrand factor (vWF) and CD31 and for the osteogenic marker osteopontin. (**A**): negative control; (**B**): hydrogel + co-culture; (**C**): hydrogel + HUVEC; (**D**): hydrogel + SDF-1. White arrows indicate positive stainings. Scale bars represent 300 µm.

In order to demonstrate osteogenic and angiogenic markers in the area of newly formed bone, we performed immunohistological staining for the endothelial markers von Willebrand factor (vWF) and CD31, as well as for the osteogenic marker ostonectin. Figure 5 shows representative examples of these immunostaining experiments.

In the negative control (HG alone) no positive staining could be verified. In agreement with the BV/TV results above, the staining of osteopontin is extremely weak in the defect area in the co-culture compared to the HUVEC monoculture and SDF-1 groups. CD31 staining, however seems to be comparable or even higher when compared to the other two groups. Interestingly, the marker vWF was expressed highly in all groups. vWF is discussed to play an important role in angiogenesis [54]; for regenerative medicine, however, vWF alone may not be sufficient to induce vascularization and bone regeneration.

**Figure 6:**
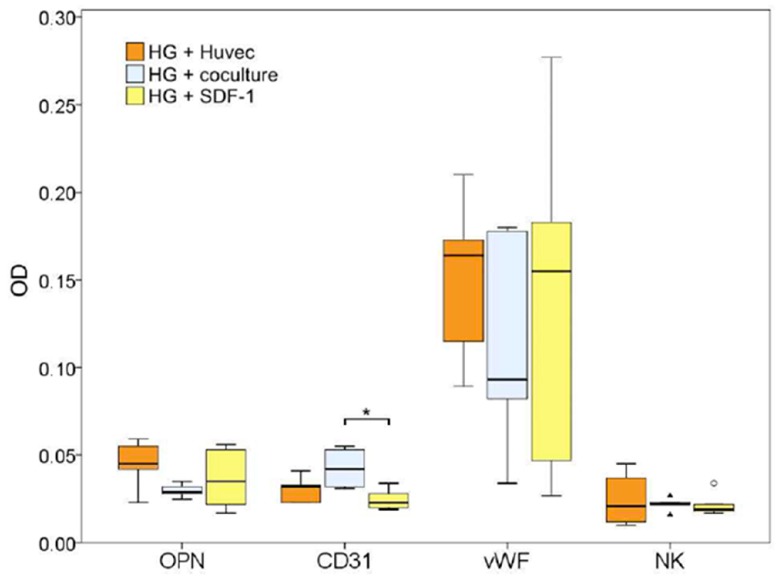
Quantification of immunohistological stainings for the endothelial markers von Willebrand factor (vWF) and CD31 and for the osteogenic marker osteopontin (OPN) in the groups HG HUVEC, HG co-culture (KOK) and HG SDF-1. NK = negative control. Significant differences are demonstrated by *, *p* ≤ 0.05.

Supporting the first indications, quantification of immunohistological staining in Figure 6 revealed a lower expression of CD31 in the group SDF-1 when compared to HUVEC and co-culture groups. This could be due to an early vascularization in the SDF-1-group. After eight weeks, this process might be completed and high concentrations of CD31 no longer verifiable. However, this has to be proven by quantification of CD31 and other endothelial markers to an earlier time point and is part of an ongoing study. In another study, SDF-1, as well as a high concentration of endothelial cells in the HUVEC group immobilized in collagen implants, lead to a fast and early vascularization by attracting endothelial progenitor cells [55] followed by neovascularization and new bone formation. After eight weeks, the process of neovascularization is almost complete and the vascularization reduces to a normal level sufficient for bone remodelling. This is in contrast to the co-culture group, where due to the relative low concentration of endothelial cells the process of neovascularization is still ongoing, as indicated by a positive CD31 staining. Osteopontin staining is highest in the groups HUVEC and SDF-1 supporting the hypothesis that in these groups bone regeneration is progressed further than in the co-culture group. However, in order to support this hypothesis, further studies, including earlier time-points to quantify vascularization as well as bone formation have to be performed and are part of the follow-up study.

## 3. Conclusions

In the presented animal study, we investigate our previously developed photocrosslinkable dextran-based hydrogels, with endotoxin concentration below the FDA limit, as carrier system for HUVEC and hOB cells or the specific growth factor SDF-1. We could demonstrate in vivo that hydrogels loaded with pro-angiogenic inducers (endothelial cells or SDF-1) enhanced bone formation significantly when compared to the negative control. Having in mind the complex procedure of cellular-based tissue engineering, further research may be focused on growth factor-induced bone regeneration, for example with dextran-based hydrogels as carrier for SDF-1. These modified dextran-based hydrogels could be used as a soft material combined with a mechanically robust, porous scaffold structure (e.g., shape-customized 3D polylactide meshes) for guided bone growth in nonunions and critical size defects. In summary, this strategy presents a promising approach to effectively address the surgical problems of failure of bone healing.

## 4. Experimental

### 4.1. Synthesis of Dextran-Based Hydrogels

Dextran hydrogel disks were prepared in 96 well plates (Greiner Bio-One, Cellstar, sterile, U-bottom) by photocrosslinking a dried polymer precursor pellet, employing the previously described benzophenone-modified dextran derivative EBP-CMD (“epoxy benzophenone-modified carboxymethyl dextran”) [21,24] as follows:

First, the 96 well plates were rinsed with ethanol, then dried and subsequently filled with the following solution (50 µL):

EBP-CMD (1.06 g) was dissolved in water (20 mL) to yield a concentration of 5% polymer stock solution. To this polymer solution an aqueous dispersion of BPTES (4-benzoylphenoxypropyl-(triethoxy)silane)-modified silica particles (32 mg/mL) was added (3.2 mL) [24,31]. For the third component, gelatin (120 mg) was dissolved in hot water (1 mL) and added to the polymer/silica nanoparticle solution.

After filling the wells with the above solution (50 µL) and drying at 37 °C, the EBP-CMD composite film was photocrosslinked by irradiation with UV light of 254 nm wavelength in a UVP CL-1000 Ultraviolet Crosslinker (UVP, LLC, Upland, CA, USA) for 60 min, which corresponds to an energy dose of 10.4 J cm^−2^. Upon exposure of the crosslinked polymer to water the network swells and forms a 3D hydrogel scaffold. 

### 4.2. Endotoxin Testing

Prior to the cell experiments, the dextran-based hydrogels were tested for endotoxin contamination, employing the endpoint chromogenic LAL (limulus amebocyte lysate) assay (Lonza, Basel, Switzerland). The assay was performed according to the manufacturer recommendations. For preparation of the endotoxin analyte solution, neat hydrogel discs were incubated in water (1 mL) directly after synthesis without preceding washing-steps for 24 h at 37 °C (conditions were transferred from ISO 10993-5: 2009; Biological evaluation of medical devices) in order to extract any potential endotoxin from the hydrogel matrix. The supernatant (50 µL) was employed in the LAL test. Furthermore, water (50 µL) from the Millipore Direct-Q UV 3 filtration device (Merck Limited, Darmstadt, Germany) before and after filter exchange was used directly for endotoxin testing. Parallel to sampling of the analyte solutions, a standard curve was established and the analysis results compared to positive controls (provided by the manufacturer within the LAL kit) and negative control (pure endotoxin free water). 

### 4.3. Immobilization of SDF-1 or BMP-7

SDF-1 (50 ng/well) or BMP-7 (50 ng/well), respectively, were entrapped in the hydrogels in presence of 1-ethyl-3-(3-dimethylaminopropyl)carbodiimid hydrochloride (EDC HCl, Applichem, Darmstadt, Germany) and *N*-hydroxysuccinimide (NHS, Sigma Aldrich, St. Louis, MO, USA) according to the following protocol. First, hydrogels were washed three times with 1× phosphate buffered saline (PBS, Dulbecco, Invitrogen, Carlsbad, CA, USA). Subsequently they were incubated in PBS for 30 min at room temperature to allow swelling and equilibration of the gels. A 0.4 M aqueous solution of EDC HCl was mixed 1:1 (*v*/*v*) with a 0.1 M aqueous solution of NHS, added directly to the hydrogels and incubated for 10 min at room temperature before SDF-1 (stock solution: 100 µg/mL in PBS, Miltenyi, Bergisch Gladbach, Germany) was added. After an incubation period of 1 h and a short rinsing step with PBS the modified gels were used for the in vivo experiments. 

### 4.4. Cell Seeding

Osteoblast cultures were prepared from cancellous bone fragments taken from patients undergoing orthopaedic and trauma surgery. Informed consent was obtained from all patients and the local ethical committee approved the investigations [No.: RLP 837.046.03(3708)]. Isolation was performed as described [56]. In short, fragments were digested with collagenase type IV (Sigma-Aldrich, St. Louis, MO, USA), washed with PBS to remove blood and fat residues and the clean bone fragments were placed in 6-well plates (Becton-Dickinson, Heidelberg, Germany) and cultured in DMEM/F12 (Biochrom, Germany) supplemented with 10% heat inactivated foetal calf serum (PAA Lab, Pasching, Austria), penicillin (100 U/mL) and streptomycin sulphate (100 mg/mL) at 37 °C, 5% CO_2_. The specific phenotype was assessed by the expression of alkaline phosphatase visualized with 5-bromo-4-chloro-3-indolyl-phosphate/nitro-blue-tetrazolium as substrate (Sigma-Aldrich, St. Louis, MO, USA) and mineralization of the extracellular matrix using Alizarin Red-S (Sigma-Aldrich, St. Louis, MO, USA) [56,57]. In order to confirm the purity of our osteoblast population, flow cytometry analyses were performed with antibodies recognizing the specific markers alkaline phosphatase (Invitrogen, Karlsruhe), osteonectin (Invitrogen, Karlsruhe, Germany) and osteopontin (Abcam, Cambridge, UK). 

### 4.5. Human Umbilical Vein Endothelial Cells (HUVEC)

HUVEC were purchased from PromoCell GmbH (Heidelberg, Germany) and cultured in EBM-2 (Endothelial Cell Growth Medium, Lonza, Walkersville, MD, USA), supplemented with the provided kit, as well as with penicillin (100 U/mL) and streptomycin sulphate (100 mg/mL), in a humidified atmosphere (5% CO_2_, 37 °C). Media was changed twice a week. Cells up to passage eight were used for experiments.

### 4.6. Mono- and Co-Culture on Dextran Hydrogels

Before cell seeding, the hydrogel films in the 96 well plates (Corning Costar, Fisher Scientific, Waltham, MA, USA) were washed three times with sterile PBS (Dulbecco, Invitrogen) to remove potential chemical residues that remained from hydrogel synthesis. After the last washing step, hydrogels were incubated for at least 30 min with PBS at room temperature to allow swelling equilibration. The PBS was removed and cells were seeded in HUVEC monoculture (100,000 cells) or co-culture from HUVEC and hOB (50,000 cells each). The incubated hydrogels were cultured for 48 h before they were implanted into mice. Monocultures were kept in their described culture medium, for the co-culture the respective media were mixed 1:1 in accordance to the cell ratio. 

### 4.7. Animal Model

Our study was approved by the local regional animal welfare committee (Landesuntersuchungsamt Rheinland-Pfalz 23 177-07/G 14-1-046). Due to xenogenic transplantation of human-derived cells, athymic nude mice were used in this investigation to avoid any species-dependent immune response reactions to these cells.

### 4.8. Hydrogel Implantation

40 five-weeks-old athymic mice (Janvier, France) were subdivided into five groups according to Table 2. One mouse of the group “co-culture” died directly after surgery due to accidental disruption of the dura mater.

Mice were anesthetised with an intra-peritoneal injection of midazolam (2 mg/kg), medetomidin (1 mg/kg) and fentanyl (5 μg/kg). After skin incision over the median sagittal area of the scalp, the calvarium was exposed and two 2.7 mm diameter critical size defects (CSD) were created bilaterally in parietal bone under low speed drilling (rotating knife, Hager & Meisinger GmbH, Neuss, Germany). Saline irrigation ensured that the dura mater and superior sagittal sinus were not damaged. The different hydrogel-type discs were implanted into the CSD and the skin was closed. Post-operative analgesia consisted of drinking water containing Tramadol (2.5 mg/100 mL). Eight weeks postoperatively the mice were sacrificed by exposure to CO_2_. After decapitation, the cavarial specimen was placed in 4.5% formaldehyde solution for subsequent radiographic and histological analysis.

### 4.9. Radiographic Analysis

Bone formation was evaluated using a high-resolution micro computed tomography (μCT) scanner (μCT 40, SCANCO Medical AG, Brüttisellen, Switzerland).

Radiographs of the calvaria were performed with the following specifications: The specimen tube of the μCT scanner containing the calvarial specimen, respectively, was run with an X-ray tube 70 kV and 113 µA and the operational resolution was 30 µm. Per rotation, 1000 pictures were taken with 1200 × 1200 pixel. Reconstruction resulted in 208 pictures/probe with a voxel size of 30 µm corresponding to a layer thickness of 30 µm and height of 6 mm. Thereby the complete calvaria could be reconstructed.

The generated graphical material was exported as common DICOM images. It was edited and analysed with the open source software “ImageJ” including the plugins “BoneJ” and “Stack Alignment.” The volume fraction (BV/TV) was then computed by measuring the bone volume (BV) inside a defined cylindrical total volume (TV). The diameter of the cylinder’s base was 3 mm and the height was 2 mm. This cylinder was orthogonally projected onto the bone defect and the calculation was performed using a voxel-based algorithm by the application “volume fraction” integrated in the BoneJ plugin.

### 4.10. Histological Analysis

After preservation in formaldehyde and radiologic analysis, the skulls were decalcified using a 10% EDTA solution for at least 14 days with the solution exchanged every second day. The heads were dehydrated by the Sakura VIP E150 Tissue Processor (Sakura Finetek Germany GmbH, Staufen im Breisgau, Germany) and then embedded in paraffin wax. The resulting blocks were cut in 5 μm slices, deparaffinised and then either stained with Haematoxylin and Eosin or an immunohistological staining was performed using specific antibodies for CD31 (Abcam, Cambridge, UK) and vWF (Abcam, Cambridge, UK) and osteopontin (Invitrogen).

The histologic slides were evaluated by descriptive histology in a standardized way. The periphery and centre of the critical size defects were compared among the five groups. Specimens were described and evaluated for cellular presence, extent of ossification defect and thickness of regenerated bone.

For quantification, Images of immunohistological staining were captured with a Zeiss Axioplan microscope (Carl Zeiss AG, Jena, Germany) and Olympus camera (XC30, Olympus soft Imaging solutions GmbH, Münster, Germany). Quantitative analyses (*N* = 5 per group) occurred with FiJi (ImageJ 1.50e). DAB staining was separated via the tool ‘Colour Deconvolution’ and the H DAB vector. A circle with a 500-pixel diameter was selected to decline the region of interest (ROI). Mean intensity of ROI was measured and converted into optical density (OD). 

### 4.11. Statistical Analysis

N of each group was 8 except the co-culture group (*n* = 7) as one animal died during surgery. We analysed the measured values by expressing the mean, median and skewness of the volume fraction using SPSS Statistics. Significance was evaluated by performing a one-way analysis of variance (ANOVA) based on a post-hoc Tamhame-T2 test. A *p* value of < 0.05 was considered statistical significant. Additionally, the statistic software automatically made an adjustment for multiple testing.

## Figures and Tables

**Table 1:** Endotoxin concentration of dextran-based hydrogels and utilized H_2_O.

Sample for Endotoxin Testing	Endotoxin Concentration (EU/mL)
Dextran-based hydrogel “non-optimized procedure”	2.62
H_2_O before filter exchange	0.71
H_2_O after filter exchange	0.37
Dextran-based hydrogel after filter exchange and usage of newly purchased chemicals with optimized handling procedure	0.45

**Table 2:** Subdivision of experimental groups.

Group Name	Number Mice/Defects
Control—pure dextran-based hydrogels	8/16
HUVEC—dextran-based hydrogels with HUVEC monoculture	8/16
Co-culture—dextran-based hydrogels with co-culture of HUVEC and hOB	7/14
SDF-1—dextran-based hydrogels with immobilized SDF-1	8/16
BMP-7—dextran-based hydrogels with immobilized BMP-7	8/16
